# Methane Emission and Milk Production of Dairy Cows Grazing Pastures Rich in Legumes or Rich in Grasses in Uruguay

**DOI:** 10.3390/ani2020288

**Published:** 2012-06-08

**Authors:** Yoana Dini, José Gere, Carolina Briano, Martin Manetti, Paula Juliarena, Valentin Picasso, Roberto Gratton, Laura Astigarraga

**Affiliations:** 1 Facultad de Agronomía, Universidad de la República, Av. Garzón 780, Montevideo 12900, Uruguay; E-Mails: caro_briano@yahoo.com.ar (C.B.); vpicasso@fagro.edu.uy (V.P.); astigarr@fagro.edu.uy (L.A.); 2 IFAS, Facultad de Ciencias Exactas, UNCPBA, CONICET, Pinto 399, Tandil 7000, Argentina; E-Mails: jgere@exa.unicen.edu.ar (J.G.); pjuliarena@exa.unicen.edu.ar (P.J.); rgratton@rec.unicen.edu.ar (R.G.); 3 INFQC, Universidad de Córdoba, CONICET, Av. Haya de la Torre s/n, Ciudad Universitaria, Córdoba 5000, Argentina; E-Mail: mmanetti@fcq.unc.edu.ar

**Keywords:** dairy cows, grazing, intake, digestibility, methane, SF_6_

## Abstract

**Simple Summary:**

GHGs emissions are relevant in evaluating environmental impact of farming systems. Methane (CH_4_) produced by enteric fermentation accounts for half of all anthropogenic emissions of GHGs in Uruguay, where ruminant production is based on year round grazing of forages. Here we compared milk production and CH_4_ emissions by dairy cows grazing two contrasting mixed pastures (rich in legumes or rich in grasses) using the SF6 tracer technique adapted to collect breath samples over 5-days periods. There were no differences in milk or CH_4_ production between the contrasting pastures, probably because of the high herbage allowance that enabled selective grazing by cows.

**Abstract:**

Understanding the impact of changing pasture composition on reducing emissions of GHGs in dairy grazing systems is an important issue to mitigate climate change. The aim of this study was to estimate daily CH_4_ emissions of dairy cows grazing two mixed pastures with contrasting composition of grasses and legumes: L pasture with 60% legumes on Dry Matter (DM) basis and G pasture with 75% grasses on DM basis. Milk production and CH_4_ emissions were compared over two periods of two weeks during spring using eight lactating Holstein cows in a 2 × 2 Latin square design. Herbage organic matter intake (HOMI) was estimated by chromic oxide dilution and herbage organic matter digestibility (OMD) was estimated by faecal index. Methane emission was estimated by using the sulfur hexafluoride (SF6) tracer technique adapted to collect breath samples over 5-day periods. OMD (0.71) and HOMI (15.7 kg OM) were not affected by pasture composition. Milk production (20.3 kg/d), milk fat yield (742 g/d) and milk protein yield (667 g/d) were similar for both pastures. This may be explained by the high herbage allowance (30 kg DM above 5 cm/cow) which allowed the cows to graze selectively, in particular in grass sward. Similarly, methane emission expressed as absolute value (368 g/d or 516 L/d) or expressed as methane yield (6.6% of Gross Energy Intake (GEI)) was not affected by treatments. In conclusion, at high herbage allowance, the quality of the diet selected by grazing cows did not differ between pastures rich in legumes or rich in grasses, and therefore there was no effect on milk or methane production.

## 1. Introduction

The growing global concerns on climate change among other environmental issues have moved researchers and farmers to include environmental impacts together with productivity when evaluating and optimizing farming systems, as reported by Johnson *et al*. [1], Pinares-Patiño *et al*. [2], Johnson *et al*. [3] and Vlaming *et al*. [4]. In particular, reducing emissions of GHGs in livestock production is a global priority to mitigate climate change, and a top priority concern for countries with grazing systems. Uruguayan ruminant production systems are predominantly pasture-based with approximately 75% of agricultural land within Uruguay dedicated to pasture. As a result of its relatively high ruminant population, enteric methane (CH_4_) emissions contribute to approximately 50% of Uruguay’s total greenhouse gas emissions as CO_2_ equivalents according to the National Greenhouse Gases (GHG) Inventory [5]. Therefore, estimating GHGs emissions is especially important for Uruguay. 

To combine sustainable dairy production and environmental conservation, the quantity and quality of forage given to dairy cattle should help mitigate methane emission. Thus, the potential methane production of forage species and pasture composition needs to be evaluated. Animals fed with legumes had a lower proportion of energy lost to methane than those fed with grasses according to Waghorn *et al.* [6] because legumes promoted higher intakes and production of the animals. Harris *et al.* [7] showed that increasing the white clover content of a pasture diet from 20% to 50% increased the dry matter intake and milk yield of cows by 13 and 32% respectively. Mixed legume-grass pastures are the basis of dairy production in Uruguay and therefore increasing the proportion of legumes in the diet of grazing animals could be a practical way to reduce national methane emissions, as well as improving livestock performance.

Measurement of methane emissions due to enteric fermentation must be taken under conditions as close as possible to typical as found in farming systems, which in Uruguay are characterized by free grazing. For this study, we used the sulfur hexafluoride (SF_6_) tracer technique reported by Johnson *et al.* [8] adapted to collect breath samples across periods of five days (multi-day sampling), instead of the original 24 h sampling. For the air sampling, we used 0.5 L stainless steel collecting vessels, where the air inflow is regulated by a very low hydraulic ball-baring conductance restrictor recently reported by Gere and Gratton [9]. Multi-day sampling favors animal welfare, simplifies logistic at the field, and reduces the number of the samples to be analyzed. This work is one of the first applications of the extended sample period version of the tracer technique for freely grazing cows. So, the main objective of this study was to estimate, through the SF_6_ tracer technique adapted to multi-day sampling, daily methane emissions of lactating dairy cows, grazing pastures with contrasting legume content.

## 2. Materials and Methods

### 2.1. Experimental Treatments and Design

The experiment was carried out in the “Centro Regional Sur”, Experimental Station of the Faculty of Agronomy (34°36'S, 56°13'W), of the Universidad de la República (Canelones, Uruguay) during the spring, from 17 October to 27 November 2010.

Treatments consisted of two pastures with contrasting composition: one with 60% of herbage mass of the legumes *Medicago sativa *L. (lucerne) and *Trifoliumrepens* L. (white clover) and 40% of the grass *Bromus auleticus* Trin. ex Nees (“cebadilla”), referred to as Legume sward hereafter, and the other pasture with 24% herbage mass of the legume *Lotus corniculatus *L. (birds-foot trefoil) and 76% of grass *Lolium multiflorum* Lam. (ryegrass), referred to as Grass sward hereafter.

A 2 × 2 Latin square design was used to evaluate the two treatments. Eight lactating Holstein cows were used over two periods of 21 days (with seven days of dietary adaptation and 14 days of faeces collection and methane measurements). The animals were allotted to two groups of four cows with similar pre-experimental milk production (24.9 ± 4.15 kg/d milk), live weight (536 ± 18 kg) and lactation stage (195 ± 7 days). Each lot was randomly assigned to the treatments in the first period, and then assigned to the other treatment in the second period. Swards were strip-grazed at a daily minimum amount of 30 kg DM/cow/day (above 5 cm). This herbage allowance was not limiting for herbage intake, according to previous work from Peyraud *et al*. [10]. A new area of pasture was offered to the cows once a day after the morning milking. Back-grazing was prevented by electric fencing. Daily areas to be offered were determined by estimating forage availability as described below.

### 2.2. Herbage Measurement

Pre-grazing herbage mass and mean sward height were measured on days 9, 12, 16 and 19 in each plot. Herbage mass was estimated by harvesting three diagonal strips (10 m × 0.5 m) with a motor scythe at a cutting height of 5 cm. Herbage mass below the motor scythe cutting height was determined by two samples cut within the frame of a 0.3 m × 0.3 m quadrat on each strip. Herbage samples were weighed fresh, sampled, and approximately 500 g was dried at 60 °C for 48 h for DM determination and for subsequent chemical analysis of forage offered. Each time, 30 tillers were taken at random and the extended height was measured to estimate the mean sward height before grazing. The same procedure was followed for determination of post-grazing herbage mass and sward height after grazing, on days 11, 14, 18 and 21. Total herbage mass to ground level was calculated as the sum of herbage mass measured above and under the motor scythe cutting height. Herbage utilization was calculated as the difference between herbage mass before grazing and after grazing and expressed as a percentage of herbage mass before grazing. The mean depth of defoliation was estimated by the difference between the values of sward height before and after grazing. 

On the same days as for the determination of pre-grazing herbage mass, three handfuls of herbage were cut at ground level with scissors on the edge of each strip, to determine the proportion of legume and grass of the herbage offered (three samples per strip and three strips per treatment). The three handfuls of herbage were bulked (one sample per strip) and arranged in a bag to keep the sward structure unaltered and immediately frozen. The proportion of legume and grass in the total DM herbage mass was determined by manual separation from a first sub-sample prior to drying. These fractions were then dried at 60 °C over 48 h and then weighed dry. The second sub-sample, with its original structure still preserved, was cut at a height corresponding to the mean post-grazing sward height. The upper portion, considered as representative of the defoliated herbage, was dried before chemical analysis. 

### 2.3. Digestibility Trial

Simultaneously to the experiment with dairy cows (days 7 to 21), the digestibility of the offered herbage (cut above motor scythe height) was measured at each experimental period with twelve Corriedale wethers (six per treatment) to characterize herbage quality. The animals were kept in metabolism crates with free access to water. Each of the two experimental periods for the digestibility trial comprised an adjustment period of 10-days, followed by 5-days dedicated to collection of faeces, orts, and feeds. For the adjustment period, feed was available *ad libitum* until DM intake stabilized (at a level of an average refusal of about 10% of the feed offered). During the five faecal collection periods, feed offered and refused, and faeces of each animal were weighed daily, and samples taken for chemical analysis.

### 2.4. Measurements on Dairy Cows

Individual herbage OM intake was determined using chromic oxide (Cr_2_O_3_) to estimate faecal organic matter output, and Nitrogen (Nf) and acid detergent fiber (ADFf) contents in the faeces (g/kg OM) to estimate herbage OM digestibility (OMd) of herbage, according to the equation established by Comeron and Peyraud [11] using herbage-based diets without supplements: 

                        OMd = 0.791 + 0.0334 Nf – 0.0038 ADFf, (R^2^ = 0.89; RSD = 0.013)

Concentrate pellets containing chromic oxide (*ca.* 20 g Cr_2_O_3_ kg of supplement) were supplied in two equal portions of 150 g each, at milking, starting on day one of the experimental period to achieve a ruminal balance (steady state). Faeces were rectal-sampled after morning milking and after evening milking from days 8 to 21, and oven dried at 60 °C over 96 h in order to measure the dry matter, the chrome concentration, and the chemical composition.

The CH_4_ emission was measured using the SF_6_ tracer technique reported by Johnson *et al.* [8]. Seven days before the beginning of the experiment, a SF_6_ permeation tube (PT, provided by the NIWA, National Institute of Water and Atmospheric Research, New Zealand) was introduced *per os* into the rumen of each animal. The permeation rates were 6.422 ± 0.416 mg/d (mean and standard deviation, respectively), as given by a 6-weeks calibration assay. The breath gas sampling system consisted of a 0.5 L stainless steel collecting vessel (canister), a ball-baring inflow restrictor adjusted to accumulate 0.5 bar of air sample during a 5-day period and a short tube used to connect both. The inflow restrictor was located just above the animal’s nostrils and protected against water and dust by means of a double filter reported by Gere and Gratton [9]. Two collecting canisters were fitted to each animal’s head by means of especially designed halters. Immediately prior to the sampling period, each collecting canister was evacuated (<0.5 mb) after cleaning with high purity nitrogen gas (N_2_). The breath gas samples were measured over two sub-periods of five days during each period of measurements (on days 10 to 14 and 16 to 20). At the end of the first 5-day sub-period, the set of canisters (two per animal) were replaced by another similar set. So at the end of each period, two sets of canisters per animal were collected across two sub-periods of five days. Additionally, an identical set as used with the cows was used to collect background air samples during each 5-day sub-period. The breath gas samples collected were analyzed immediately after the end of the experimental period. Daily CH_4_ emissions were calculated from SF_6_ release rate and CH_4_/SF_6_ ratio of concentrations in breath samples, after correction for background gas concentrations [8]. 

The cows were milked twice, from 6:00 to 6:30 h in the morning and from 17:00 to 17:30 h in the afternoon. Individual milk production was measured each day. Milk fat and protein contents were determined on four consecutive days, twice in each experimental period (on days 9 to 12 and 16 to 19). Cows were weighed on the last day of each experimental period.

### 2.5. Chemical Analysis

All the dried samples were ground through a 1 mm screen before chemical analysis. The dry matter (DM) concentration was determined by drying at 105 °C in an oven for 24 h and ash content was determined by incineration at 600 °C for 4 h for organic matter (OM) calculation. Crude protein (CP) content was determined using the Kjeldahl method (7.021 procedure) in A.O.A.C [12]. Content of a neutral detergent fiber (NDFom) was determined without sodium sulfite and with heat stable amylase. Acid detergent fiber (ADFom) and acid detergent lignin (lignin) were determined using sequential analysis and are expressed exclusive of residual ash according to Van Soest *et al*. [13]. An Ankom apparatus (Ankom 220, Fairport, NY, USA) was used for extraction and filtering. Condensed tannins were analyzed according to Schofield *et al.* [14]. Gross Energy (GE) was determined using an adiabatic bomb calorimeter (Gallenkamp Autobomb; Loughborough, Leics, UK).

Milk samples at every milking of each collection period were analyzed for fat, protein, and lactose content with infrared spectroscopy (Bentley 2000 Infrared Milk Analyzer, Bentley Instruments Inc., Chaska, MN, USA). Chromium (Cr) concentration in faecal samples was determined by atomic absorption spectrophometry (Perkin-Elmer 2380, Norwald, CT, USA), using air and an acetylene flame according to William *et al*. [15]. Cr standards were prepared using pre-trial faecal collections that contained no Cr. 

The concentrations of CH_4_ and SF_6_ were determined by chromatography in a specialized Laboratory (INFQC, Universidad de Córdoba, CONICET, Córdoba, Argentina). The samples were injected at once in two different setups. For CH_4_ a 3 mL loop was used, a HP-PLOT Q column and a FID detector. For SF_6_ a 10 mL loop, a HP-MOLSIV column and an ECD detector. Each sample was analyzed at least twice and the average values were used to obtain methane concentration and methane emission. Maximum delay between the collection and the determination of CH_4_ and SF_6_ concentrations was 15 days. 

### 2.6. Statistical Analysis

All the animal data were analyzed according to a 2 × 2 Latin square design, using PROC MIXED function of SAS (version 9.1; SAS Inst. Inc., Cary, NC, USA) using the model: Y = μ+ Pi + Tj + Ak + εijk, where μ was the overall mean, Pi was the fixed effect of period (i = 1 to 2), Tj was the fixed effect of sward (j = 1 to 2), Ak was the random effect of animal (k = 1 to 8) and εijk was the associated error. The production and composition of the milk were analyzed as repeated measures over time, according to an autoregressive model of order one reported by Littel *et al.* [16]. The pasture data were analyzed according to a 2 × 2 Latin square design by ANOVA using the GLM procedure of SAS (SAS Institute, Inc., 2001) with the following model: Y = μ+ Pi + Tj + εij. In both MIXED and GLM models initially the interaction treatment x period was included, and it was not significant, therefore, following the recommendations of Lorenzen and Anderson [17] it was not included in the final model. In order to compare the mean treatment values, the test of the minimum significant difference was used.

## 3. Results and Discussion

### 3.1. Sward Characteristics and Herbage Defoliated

Total rainfall (186 mm) and mean temperature (15.5 °C) in the spring 2010 were below seasonally climatic conditions (309 mm, and 20.0 °C, mean over 10 years). 

Total herbage mass was lower on the Grass sward (−869 kg DM/ha) (*P* = 0.0002). However, herbage mass above 5 cm (2,165 kg DM/ha on average) and sward height (29.5 cm on average) were similar between pastures (Table 1). Botanical composition of both swards was significantly different as intended (*P* < 0.0001): Legume sward presented a higher proportion of legumes (60% of herbage mass above 5 cm as *Medicago sativa *and *Trifoliun repens*) while Grass sward presented a higher proportion of grasses (76% of herbage mass above 5 cm as *Lolium multiflorum*). Both swards were at reproductive stage during the experiment.

Chemical composition (above 5 cm) was also different between swards. Grass sward had higher DM content (*P* = 0.0008), aNDFom content (*P* = 0.0290), and ADFom content tended to be higher too (*P* = 0.0618), while CP content was two times lower (−102 g/kg DM) (*P* < 0.0001) compared to Legume sward. Gross energy was also lower in Grass sward (*P* = 0.0017). Condensed tannins were higher on Legume sward than on Grass sward (+1.8 g /kg DM, *P* = 0.0205), and for both pastures, these values were slightly higher than those reported by Barry and McNabb [18] for lucerne and ryegrass forage.

The *in vivo* digestibility trial resulted in higher DM (+73 g/kg DM, *P* = 0.0006), OM (+39 g/kg DM, *P* = 0.0103), and aNDFom (+67 g/kg DM, *P* = 0.00016) digestibility for the Legume sward than the Grass sward. However, the digestibility of ADFom was similar for both swards (*P* = 0.1516) (Table 1). In particular, the DM digestibility of the Grass sward is similar to values reported by Molano and Clark [19] for reproductive ryegrass in an experiment to determine the effect of quantity and quality of forage intake on CH_4_ emissions on wether lambs.

**Table 1:** Biomass, height, botanical, chemical composition, and *in vivo* digestibility of the two experimental swards and significance (*P*).

	Pasture treatment		
Sward parameters	Legume	Grass	*P*
Herbage mass (kg DM/ha)	4,335	3,499	0.0002
Herbage mass above 5 cm (kg DM/ha)	2,309	2,021	0.2201
Sward height (cm)	31	28	0.5153
			
*Botanical composition * ^1^			
Grass/Legume ratio (%DM)	40/60	76/24	<0.0001
			
*Chemical composition *^1^ (g/kg DM)			
DM (g/kg)	276	384	0.0008
OM (g/kg DM)	920	903	0.0461
CP	204	102	<0.0001
Condensed tannins	4.6	2.8	0.0205
aNDFom	469	540	0.0260
ADFom	265	312	0.0618
Lignin (sa)	76	62	0.1149
GE (kJ/kg DM)	18.1	16.7	0.0017
			
*In vivo digestibility *^1^ (g/kg)			
DM	689	616	0.0006
OM	698	659	0.0103
aNDFom	633	566	0.0016
ADFom	525	489	0.1516
^1^ Above the motor scythe cutting height (5 cm).			

Cows on the Grass sward exhibited a higher herbage allowance than in the Legume sward (+10 kg DM/cow/day, *P* = 0.0226), as the area allocated per cow was higher on Grass sward (Table 2). However, in both treatments herbage allowance was above the stipulated minimum amount of 30 kg DM/cow/day (above 5 cm) which allows an *ad libitum* intake according to Peyraud *et al*. [10]. The depth of defoliation (9.5 cm on average) and the herbage utilization (42% on average) were similar for both swards. 

Due to the high post grazing sward height, the chemical composition of defoliated herbage on Grass sward showed a different composition from the offered herbage (Table 2). On the Grass sward the grazed forage contained more CP (+31 g/kg DM), more GE (+2.3 kJ/kg DM), less aNDFom (−58 g/kg DM), and ADFom (−55 g/kg DM) than on the offered sward above 5 cm (Table 2). These results are in agreement with the large vertical gradients observed in the different chemical constituents from the upper to the lower layer of a ryegrass sward reported by Delagarde *et al.* [20], The content of condensed tannins of the herbage defoliated was similar for both pastures (*P* = 0.5119).

**Table 2:** Herbage allowance, depth of defoliation, herbage utilization and chemical composition of the herbage defoliated by grazing dairy cows on pastures rich in legumes or rich in grasses.

	Pasture treatment		
	Legume	Grass	*P*
Herbage allowance ^1^^,^^2^ (kg DM/ cow/day)	35	45	0.0226
Depth of defoliation (cm)	11	8	0.6436
Herbage utilization ^1^ (%)	46	37	0.2650
			
*Chemical composition *^1^ (g/kg DM)			
DM (g/kg)	894	898	0.4399
OM	923	926	0.4481
CP	180	133	0.0225
Condensed tannins	2.2	2.5	0.5119
aNDFom	464	482	0.6946
ADFom	264	259	0.8578
Lig (sa)	47	38	0.0582
GE (kJ/kg DM)	18.7	19.0	0.5954

^1^ Above the motor scythe cutting height (5 cm). ^ 2^ Area allocated per cow: 150 and 225 m^2^ on L and G sward respectively

As observed in the offered grass, the defoliated Grass sward had a lower CP content than defoliated Legume sward (−47 g/kg DM, *P* = 0.0225). Nevertheless, the CP content of the Grass sward is above the minimum critical content for ruminal digestion, according to Poppi and McLennan [21] (12.5% DM for pastures ranging on 62% DM digestibility). The lower CP content of defoliated herbage in Legume sward compared to the offered herbage may be explained by a lower proportion of white clover, because the post-grazing height (20 cm) was above the white clover height.

### 3.2. Herbage Intake at Grazing

Faecal OM output was similar for Legume and Grass treatments (4.5 kg OM/cow on average). The OM digestibility of defoliated herbage, estimated by faecal index according to Comeron and Peyraud [11], did not differ between treatments (707 g/kg OM on average) and was higher than values measured on the digestibility trial with wethers on the offered herbage (above 5 cm). Due to the high herbage allowance per cow and the relatively high levels of herbage height in both swards, the post grazing height remained substantially higher (20 cm on average) than the cutting height of the motor scythe (5 cm on average), allowing the cows to consume a higher quality of herbage, in particular in Grass sward as discussed above (Table 2). Daily herbage OM intake was similar among treatments and averaged 15.7 kg OM/cow (Table 3), which agrees with the calculated amount of defoliated herbage presented in Table 2 (herbage allowance x herbage utilization). Therefore, the early stage of heading in ryegrass did not reduce herbage OM intake as reported by Astigarraga and Peyraud [22]. Finally, digestible OM intake was also similar (11.2 kg DOM/cow on average) among swards. The digestible OM intake is in accordance with the requirements for maintenance, milk production, and live weight gain calculated from the standards given by INRA [23] and adjusted for the energy expenditure used for muscular activity while grazing according to Ribeiro *et al.* [24], indicating that any bias in the estimation of OM intake was not significant.

**Table 3:** Effect of pastures rich in legumes or rich in grasses on faecal output, herbage organic matter digestibility and organic matter, dry matter and total digestible organic matter intake by grazing dairy cows.

	Pastures treatment		
	Legume	Grass	*P*
Faecal output (kg OM/d)	4.5	4.6	0.8476
Herbage OM digestibility (g/kg OM)	711	704	0.1996
Herbage OM intake (kg/cow/day)	15.9	15.5	0.7558
Herbage DM intake (kg/cow/day)	17.3	16.8	0.7556
Total digestible OM intake (kg/cow/day)	11.4	10.9	0.6191

### 3.3. Milk Production and Composition, and Live Weight Variation

Milk yield (20.3 kg/cow on average) and milk composition (37.1 and 33.3 g/kg milk for fat and protein contents respectively, on average) were not affected by the experimental pastures (Table 4). Live weight variation (+16 kg LW on average) was also not affected by treatments. These results are clearly associated with a similar total DOM intake and thus in digestible energy intake reported above.

**Table 4:** Effect of pastures rich in legumes or rich in grasses on milk yield, milk composition and live weight variation of grazing dairy cows.

	Pastures treatment		
	Legume	Grass	*P*
Milk yield (kg/cow/day)	20.7	19.9	0.7508
Fat content (g/kg)	37.7	36.5	0.5030
Fat yield (g/day)	772	711	0.3630
Protein content (g/kg)	34.0	32.5	0.1068
Protein yield (g/day)	699	636	0.3728
Fat corrected milk (FCM 4%) (kg/cow/day)	19.9	18.7	0.5173
Live weight variation (kg/cow)	+12	+20	0.2317

### 3.4. Methane Emission and Methane Yield

The efficiency of sample collection was 94% (30 successful samples were collected from 32 expected). The methane emission (g/d and L/d) was similar among treatments, and averaged 368 g/d (Table 5). The CV for absolute CH_4_ emission was 13.8%. 

**Table 5:** Effect of pastures rich in legumes or rich in grasses on methane emission and methane yield of grazing dairy cows.

	Pastures treatment		
	Legume	Grass	*P*
Methane emission (g/cow/day)	364	372	0.7237
Methane emission (L/cow/day)	510	521	
Methane emission (g/kg FCM 4%)	18.6	20.6	0.3517
Methane yield			
as Gross Energy intake (Y_m_)	6.4	6.7	0.5971
as g methane/kg DMI	21.6	22.7	0.5821

Methane yield per unit of DMI (22.2 g/kg on average) and as a percentage of gross energy intake (GEI) (Ym = 6.6% on average) did not differ between treatments, likely because DM intake and ingested energy were similar for Legume and Grass swards in this study (Table 3). These values are in the range reported in New Zealand by Lassey [25] and Boadi *et al.* [26] for dairy cattle grazing on temperate forages. Ramirez-Restropo and Barry [27], suggest that feeding forage legumes like lucerne or red clover tends to decrease CH_4_ losses (g/kg DMI) compared to grass. Nevertheless, the results of our study do not seem to confirm what. Hammond *et al.* [28,29] recently suggested that methane emissions could be more related to DM intake, which allows variations in the composition of the diet selected at grazing, in particular for Grass swards. In fact, the methane emission and the methane yield (as g/kg DMI or Ym) reported by Lee *et al.* [30] on dairy cows fed increasing proportions of legume (white clover), are closer at similar DMI than for similar proportions of legume in the forage mixture. Archimède *et al.* [31] found no differences in methane per kg OM intake between C3 grasses and cold legumes in a meta-analysis compared for the same fiber content, digestibility and intake. Finally, methane emission expressed by unit of milk production was similar between treatments, as no differences in milk performance were observed. 

## 4. Conclusions

The estimation of methane emission under grazing conditions requires taking into account selective grazing, and consequently, determining the quality of the herbage actually defoliated by grazing animals. This study shows that at a high herbage allowance, the quality of the diet selected by grazing cows did not differ between a pasture rich in grasses and a pasture rich in legumes and as a result, methane emission expressed per unit intake (DM or OM) was similar for both swards. These results raise the issue that differences between types of pastures are more related to differences in quality than in other inherent properties. Additionally, the multi-day sampling version of the SF_6_ tracer technique used in this experiment resulted in values of methane emission in good agreement with those reported in the International Bibliography for Dairy Cows where the single-day sampling version of the tracer technique was employed, which may thus enable a valuable simplification of the experimental logistics.
